# Combination Chemotherapy for Influenza

**DOI:** 10.3390/v2081510

**Published:** 2010-07-27

**Authors:** Elena A. Govorkova, Robert G. Webster

**Affiliations:** Department of Infectious Diseases, Division of Virology, St. Jude Children’s Research Hospital, 262 Danny Thomas Place, Memphis, TN 38105-3678, USA; E-Mail: elena.govorkova@stjude.org

**Keywords:** combination therapy, antivirals, influenza virus, neuraminidase inhibitors, oseltamivir, amantadine, ribavirin

## Abstract

The emergence of pandemic H1N1 influenza viruses in April 2009 and the continuous evolution of highly pathogenic H5N1 influenza viruses underscore the urgency of novel approaches to chemotherapy for human influenza infection. Anti-influenza drugs are currently limited to the neuraminidase inhibitors (oseltamivir and zanamivir) and to M2 ion channel blockers (amantadine and rimantadine), although resistance to the latter class develops rapidly. Potential targets for the development of new anti-influenza agents include the viral polymerase (and endonuclease), the hemagglutinin, and the non-structural protein NS1. The limitations of monotherapy and the emergence of drug-resistant variants make combination chemotherapy the logical therapeutic option. Here we review the experimental data on combination chemotherapy with currently available agents and the development of new agents and therapy targets.

## 1. Introduction

Influenza continues to cause an unacceptable number of deaths and substantial economic losses worldwide. Although vaccination strategies are the mainstay of influenza control and prevention, the efficacy of this approach can be limited by the four-to-six-month minimum time required to produce a specific vaccine against a newly emergent virus, a poor match between the vaccine and the circulating strain, and poor immune responses among elderly patients [1]. Influenza virus infection can be effectively treated with anti-influenza antiviral agents [2,3], as it was during the rapid emergence of the pandemic H1N1 influenza virus in 2009 when no vaccine was available. At present, two classes of antiviral drugs are approved for influenza therapy: M2 ion channel blockers (oral amantadine and its derivative rimantadine) and neuraminidase (NA) inhibitors (oral oseltamivir and inhaled zanamivir). These agents either prevent viral uncoating inside the cell (M2-blockers) or prevent the release of progeny virions from infected cells (NA inhibitors). The development of resistance is a major obstacle to the usefulness of both classes of anti-influenza agents, which could provide a growth advantage to variants carrying drug-resistance mutations, more likely under drug selective pressure. The emergence of pathogenic, transmissible, amantadine-resistant H1N1 and H3N2 seasonal influenza variants, amantadine resistance in the pandemic H1N1 2009 influenza viruses, and a significant increase in amantadine resistance among some highly pathogenic H5N1 viruses, have limited the usefulness of M2 ion channel blockers [4–7]. The NA inhibitor resistance-associated mutations in influenza viruses are drug-specific and NA subtype-specific [8]. In experimental animal models, it was shown that the NA inhibitor resistant variants differed in fitness and transmissibility; this difference may be influenced by the location of NA mutation(s) [9–11]. These findings indicated that transmissible NA inhibitor resistant variants may emerge, and therefore continuous monitoring for NA inhibitor resistance is necessary. Until 2007, influenza viruses resistant to the NA inhibitor oseltamivir were isolated at a low level. The incidence of oseltamivir resistance seen in clinical trial samples was 0.33% (4/1228) in adults/adolescents (≥13 years), 4.0% (17/421) in children (1 ≤ 12 years) and 1.26% overall [12]. However, during the 2007–2008 influenza season, oseltamivir-resistant variants with the H275Y NA amino acid substitution (N1 numbering) became widespread, first in the Northern Hemisphere [13,14] and then in the Southern Hemisphere [15]. Importantly, oseltamivir-resistant variants have rarely been reported among the novel pandemic H1N1 influenza viruses that emerged in April 2009 [16], and oseltamivir is the treatment agent favored by clinicians [17,18].

**Figure 1.**
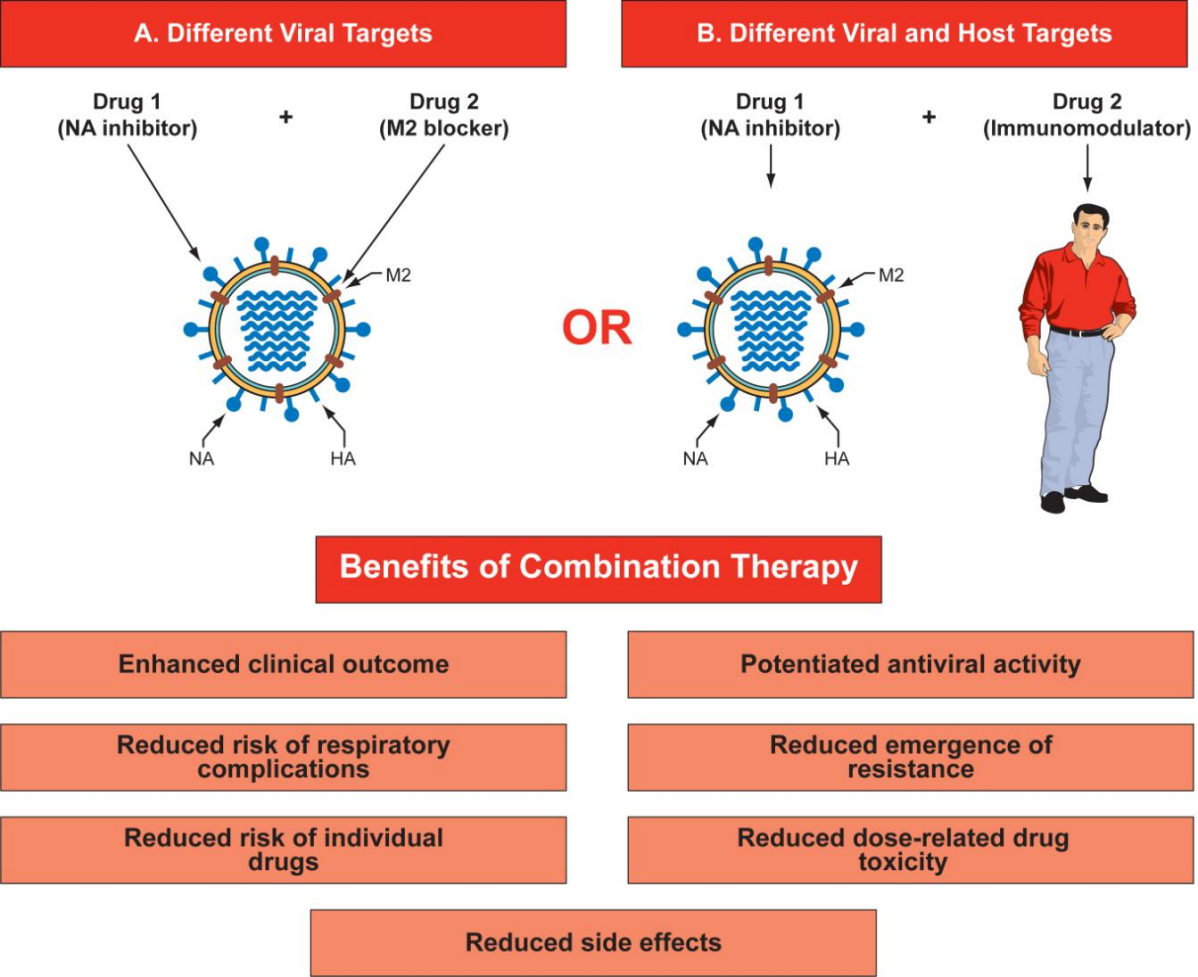
Benefits of combination therapy for influenza. Combination therapy could target different viral proteins **(A)** that have different mechanisms of antiviral action (for example, NA inhibitor and M2 ion channel blocker), or **(B)** target virus and host factors that affect virus replication and host defense mechanisms (for example, NA inhibitor and immunomodulator). This diagram represents only double drug combinations, but a multidrug approach could consist of three or more drugs in combination. Abbreviations: HA- hemagglutinin; NA - neuraminidase; M2 - matrix protein.

An attractive approach for countering drug resistance is combination chemotherapy with two or more drugs that target different viral proteins or host factors and for which the mechanisms of resistance differ. Such combination therapy may reduce the likelihood that resistance to a single drug will emerge and reduces the effect of resistance that does emerge. Moreover, combination therapy may not only potentiate antiviral activity but may also enhance clinical outcomes by allowing reductions of the doses of individual drugs, thereby reducing dose-related drug toxicity and side effects. In addition, it can reduce the risk of respiratory complications (Figure 1).

The pharmacological rationale for multiple-drug combination therapy is exemplified by combination HIV treatment regimens that keep the plasma viral load below detectable levels, prevent the emergence of resistance, and allow successful management of HIV-infected patients [19,20]. This review presents the experimental and available clinical data on combination chemotherapy for influenza and discusses several investigational and exploratory agents that may be used in combination.

## 2. Antiviral activity of drug combinations in cell culture

The idea of using combination drug regimens to control influenza virus infection is not novel. For a number of years, investigators have studied the activity of combinations of various compounds against different subtypes of influenza virus *in vitro* and *in vivo*, seeking either synergistic or additive effects. Two or more drugs are combined to achieve critical experimental and potentially clinical end-points, producing interactions defined as “synergistic,” “additive,” or “antagonistic” when their combined effect exceeds, equals, or is less than that of the sum of the effects of the individual drugs, respectively [21].

The results of various studies that have assessed the effects of the double and triple drug combinations on influenza virus infection *in vitro* are summarized in Table 1. Earlier studies showed that rimantadine combined with the synthetic nucleoside ribavirin caused additive and, at specific concentrations, synergistic inhibition of influenza A/FPV Weybridge (H7N7) virus infection in chick embryo fibroblast cell cultures [22]. Although ribavirin has been officially approved for other conditions (hepatitis C and severe respiratory syncytial virus infection), it inhibits influenza A and B virus infection *in vitro* and in animal models [23–25]. Its metabolite ribavirin triphosphate inhibits the function of virus-coded RNA polymerases, which provides broad antiviral activity [23]. Rimantadine and ribavirin combinations were reported to reduce human influenza A/Texas/1/77 (H3N2) and A/USSR/90/77 (H1N1) virus yields in Madin-Darby canine kidney (MDCK) cells more than either agent alone [26]. Human interferon-α and rimantadine or ribavirin additively or synergistically reduce the yield of clinical H3N2 or H1N1 influenza A isolates in primary rhesus monkey kidney cells [27]. Other studies have tested combination regimens that included experimental compounds, such as polyoxometalate (PM)-523 [28], infusions of the natural antiviral agent *Flos verbasci* [29], and other plant preparations [30].

Discovery of the NA inhibitors was a significant milestone in influenza antiviral therapy [31–33] and introduced new options for combination chemotherapy. The NA inhibitor zanamivir combined with rimantadine, ribavirin, or 2′-deoxy-2′-fluoroguanosine showed additive effects against influenza A viruses in MDCK cells [34], and the NA inhibitor peramivir enhanced the reduction of virus yield in MDCK cells when combined with ribavirin [35]. Combinations that paired rimantadine with an NA inhibitor (zanamivir, oseltamivir carboxylate [active metabolite of oseltamivir], or peramivir) reduced extracellular H1N1 and H3N2 influenza virus yields in MDCK cells more efficiently than any of the drugs alone [36].

A number of reports have described the activity of drug combinations against avian H5N1 influenza viruses *in vitro*. The yield of human A/Hong Kong/156/97 (H5N1) influenza virus in MDCK cells was reduced significantly more than by monotherapy (*P*<0.005) when the cells were treated with the combination of amantadine and low-dose (≤1 μM) oseltamivir carboxylate [37]. The drug pairs amantadine plus oseltamivir carboxylate and amantadine plus ribavirin acted synergistically over a range of doses against avian A/Duck/MN/1525/81 (H5N1) virus in MDCK cells [38], although this virus lacks the multibasic HA amino acid R-X-R/K-R motif required for high pathogenicity.

Most studies have tested drug combinations against influenza viruses that were sensitive to both drugs. However, taking into consideration that all seasonal H3N2 and 2009 H1N1 influenza strains are resistant to amantadine [4,5,7], and the rapid emergence and spread of oseltamivir-resistant influenza viruses in 2007–2008 season [13–15], it is important to determine the efficacy of drug combinations against drug-resistant viruses. Smee and colleagues [38] found that the addition of amantadine to oseltamivir carboxylate did not improve activity against amantadine-resistant A/Duck/MN/1525/81 (H5N1) virus in MDCK cells. The activity of a triple combination antiviral drug (TCAD) regimen (oseltamivir carboxylate, amantadine, and ribavirin) was tested against amantadine- and oseltamivir-resistant seasonal influenza viruses *in vitro* [39]. Surprisingly, amantadine and oseltamivir carboxylate contributed to the TCAD regimen’s antiviral activity against amantadine- and oseltamivir-resistant viruses at concentrations that had shown no activity in single-agent testing and that were clinically achievable [39]. The interactions between M2, HA, and NA proteins on the surface of the influenza particles are complex and not well understood. The authors suggested that as a result of protein-protein interactions between M2, HA and NA, the binding of a drug at one site may affect the confirmation and therefore affinity of the drug at another site [39].

The activity of a TCAD regimen (oseltamivir carboxylate, amantadine, and ribavirin) against H1N1 2009 pandemic (A/California/04/09, A/California/05/09, and A/California/10/09) and three other influenza viruses [(A/New Caledonia/20/99 (H1N1), A/Sydney/05/97 (H3N2) and A/Duck/MN/1525/81 (H5N1)] was assessed on the basis of cytopathic effect inhibition in MDCK cells [39,40]. Importantly, this triple combination proved to be highly synergistic, and the synergy of the TCAD regimen was significantly greater than that of any double combination tested (P < 0.05), including a combination comprising two NA inhibitors. This synergy was observed at concentrations achievable in human plasma at doses previously shown to be safe [39]; therefore, it may produce a markedly improved clinical outcome. Because combination treatment may inhibit the selection and outgrowth of drug-resistant viruses by reducing the number of replication cycles, it may also reduce the proportion of virus particles carrying resistance mutations. Ilyushina and colleagues [37] tested *in vitro* the hypothesis that combinations of amantadine and oseltamivir carboxylate can prevent or reduce the emergence of drug-resistant variants. It was shown that even low concentrations of oseltamivir carboxylate prevented the emergence of amantadine-resistant variants of the H1N1, H3N2, and H5N1 subtypes grown in the presence of the two drugs in MDCK cells [37].

An important initial step in the evaluation of combination therapy is to determine whether the combined agents reduce influenza virus replication additively, synergistically, or antagonistically. A three-dimensional approach that allows a complete analysis of all tested drug concentrations and biological effects is considered the most suitable model for analysis of drug interactions [41]. However, the available data on the synergistic interactions of different drugs are inconsistent to a degree. These differences may be attributed to the specific doses of each drug used in the studies; different influenza virus strains and passage history; different multiplicities of infection; different cell types, levels of confluence, and cell stocks; and different experimental designs. The endpoints used in studies also vary, these have included (1) inhibition of virus-induced cytopathic effects as determined by staining with neutral red, (2) inhibition of extracellular virus yields as shown by infectivity (plaque reduction assay and 50% tissue culture infectious dose [TCID_50_]) with and without drug pressure, (3) inhibition of cell-associated virus yields in MDCK cells as shown by microneutralization and subsequent enzyme-linked immunosorbent assay (ELISA), and (4) inhibition of RNA copy number. Therefore, a standard approach is needed to evaluate antiviral activity and drug interactions in *in vitro* systems. Furthermore, *in vitro* studies of multidrug regimens must be followed by animal experiments and clinical trials to define dose requirements and dose-response relationships between antiviral agents.

## 3. Antiviral activity of drug combinations *in vivo*

A number of animal studies have demonstrated the benefits of combination chemotherapy for influenza virus infection [35,38,42–45]. The experimental data on antiviral activity of double drug combinations against influenza A and B viruses in a lethal mouse model are summarized in Table 2.

The mouse model is the only animal model currently used for evaluation of antiviral drug combinations. Analysis of drug interactions requires a sufficient number of animals per group, informative parameters of evaluation (e.g., survival, weight loss, virus titers, and oxygen saturation) and a sufficient number of parameters for statistical evaluation. It is often difficult to determine drug interactions in animals because of small group sizes and the variability of effects at particular drug doses. Analysis can be complicated by variability in response rates at specific doses, in the parameter under evaluation, in the virus challenge dose, and in the timing of the initiation of antiviral treatment.

The use of M2 blockers in combination with ribavirin or oseltamivir was associated with enhanced survival and was significantly more effective than either drug alone in a mouse model [46,47]. The therapeutic synergism of aprotinin, an influenza protease inhibitor, and rimantadine successfully protected mice from a lethal challenge with a mouse-adapted influenza virus [48]. The NA inhibitor peramivir was shown to interact additively and synergistically with ribavirin to reduce A/NWS/33 (H1N1) influenza infection with no enhancement of toxicity in mice [35]. Synergistic interaction was reported when rimantadine and oseltamivir were given to mice that had been intranasally inoculated with 10 or 20 50% mouse lethal doses (MLD_50_) of A/Aichi/2/68 (H3N2) virus when the compounds were administered simultaneously for five days, starting 4 h before inoculation [42].

Laboratory mouse-adapted influenza virus strains are customarily used in these studies, and they usually do not accurately represent the viruses currently circulating in nature. However, there have been attempts to study more recent influenza strains. Two new lethal infection models in mice were developed using mouse-adapted influenza A/New Caledonia/20/99 (H1N1) and influenza B/Sichuan/379/99 viruses to study oral treatment with oseltamivir and ribavirin, both alone and in combination [25]. The activity of oseltamivir-ribavirin combinations against these viruses differed from that reported for older, more established virus strains: oseltamivir was less effective and ribavirin was more active against A/New Caledonia/20/99 (H1N1) and B/Sichuan/379/99 influenza viruses [25].

Human infection with highly pathogenic avian H5N1 influenza viruses differs from infection with seasonal human H1N1 or H3N2 viruses mainly in that the H5N1 viruses have high replication efficiency, are disseminated beyond the respiratory tract (causing multiorgan failure), and induce hypercytokinemia [49]. Although the mechanisms of these multiple pathogenic effects in humans remain to be defined, preclinical animal studies provide an excellent prototype for optimizing antiviral therapy against highly pathogenic H5N1 viruses. A significant body of information about the efficacy of drug combinations against H5N1 influenza viruses *in vivo* is available. In a mouse model, oseltamivir combined with amantadine or rimantadine was more effective than monotherapy with oseltamivir in preventing the death of mice infected with H5N1 or H9N2 viruses [50]. Combinations of amantadine and oseltamivir were tested against recombinant amantadine-sensitive and amantadine-resistant A/Vietnam/1203/04 (H5N1) influenza virus [43]. In mice lethally challenged with A/Vietnam/1203/04 (H5N1) influenza virus that was sensitive to both oseltamivir and amantadine, combination therapy produced an additive benefit: survival was 30% with oseltamivir alone, 60% with amantadine alone, and 90% with combination treatment [43]. This combination also significantly decreased viral titers in the lungs and prevented spread to the brain. However, combination therapy was no better than oseltamivir alone against the amantadine-resistant A/Vietnam/1203/04 (H5N1) influenza virus. A similar effect was reported when amantadine-oseltamivir combinations were tested against amantadine-resistant A/Duck/MN/1525/81 (H5N1) virus in mice [38]. In contrast, oseltamivir-ribavirin treatment significantly reduced the mortality of mice inoculated with A/Duck/MN/1525/81 (H5N1) influenza virus [38]. Oseltamivir and ribavirin showed principally additive efficacy against both clade 1 and clade 2 H5N1 influenza viruses. However, the efficacy of the drug combination against two H5N1 viruses differed: higher doses were required to protect mice against A/Turkey/15/06 virus than against A/Vietnam/1203/04 virus [44]. Despite the efficacy of ribavirin in these studies, its use is limited by a relatively small therapeutic index, induction of hemolytic anemia at high doses, high toxicity, and potential teratogenic effects [23]. It is still unclear whether combination therapy can produce sufficiently synergistic effects to allow a reduction of the dose of ribavirin to acceptable and safe levels. These observations highlight the need for additional antiviral agents that can be used in combination with oseltamivir for the management of H5N1 influenza virus infections.

## 4. Clinical data

Preclinical studies have demonstrated the benefits of combination therapy over monotherapy in inhibiting virus replication and reducing the risk of the emergence of resistant viruses. However, the clinical evaluation of combination therapy with currently available agents has received little attention. Controlled clinical therapy trials are impeded not only by the difficulty of organizing them but also by the limited number of antiviral drugs available for use in combinations and the high level of resistance to the adamantanes (amantadine and rimantadine) in circulating influenza strains. Several years ago, the National Institute of Allergy and Infectious Diseases (NIAID) Antiviral Study Group compared outcomes among hospitalized adults with influenza who received either nebulized zanamivir plus oral rimantadine or nebulized saline placebo plus oral rimantadine [51]. These studies were undertaken before the spread of amantadine resistance worldwide. Patients treated with zanamivir plus rimantadine demonstrated a nonsignificant trend toward fewer days of virus shedding and were less likely to have a severe cough. Moreover, no resistant variants were found in the group receiving combination therapy, while 2 of 11 patients in the rimantadine monotherapy group had resistant virus [51].

To detect unanticipated interactions of the drugs used in combinations, the pharmacokinetics of amantadine (100 mg orally twice daily) and oseltamivir (75 mg orally twice daily) administered alone and in combination for five days was evaluated in a randomized, crossover study (n = 17) [52]. The pharmacokinetics of amantadine was not affected by coadministration of oseltamivir. Similarly, amantadine did not affect the pharmacokinetics of oseltamivir or its metabolite, oseltamivir carboxylate. There was no evidence of an increase in the frequency or severity of adverse events when amantadine and oseltamivir were used in combination [52].

The pharmacokinetics and tolerability of the oseltamivir-probenecid combination were evaluated in healthy volunteers [53,54]. The two tested regimens were (1) oseltamivir 150 mg once a day and probenecid 500 mg orally four times a day for four days [53] and (2) 75 mg of oral oseltamivir every 48 h and 500 mg of probenecid four times daily for 15 days [54]. Subjects who received both oseltamivir and probenecid had an oseltamivir carboxylate plasma concentration 2–2.5 times that of subjects who received oseltamivir alone. Probenecid inhibits renal tubular urate resorption and reduces the excretion of several medications [55,56]. Therefore, the oseltamivir-probenecid combination must be used with caution in patients receiving renally excreted medications.

Ongoing NIAID efforts to conduct clinical studies of combination anti-influenza regimens will provide much-needed information on the prospects of this approach for the treatment of influenza.

## 5. Investigational antiviral targets and agents

**Figure 2.**
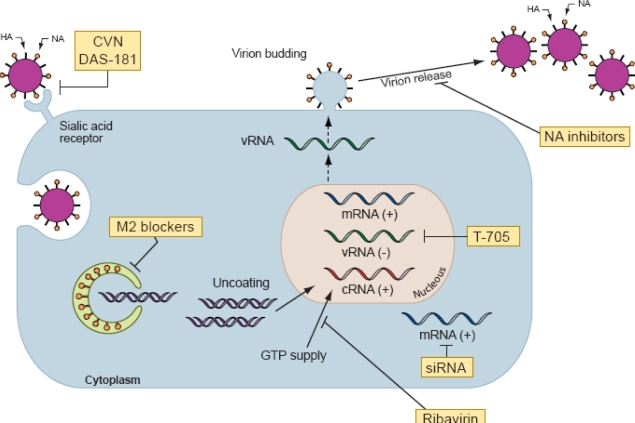
Schematic representation of influenza virus replication cycle and sites of action of antiviral agents. **Note.** Influenza A virus contains eight RNA segments of negative polarity coding for at least 11 viral proteins. The surface proteins of influenza A virus consist of two glycoproteins, hemagglutinin (HA) and neuraminidase (NA), and the M2 protein. The HA protein attaches the virus to sialic acid–containing receptors on the cell surface and initiates infection. A fusion protein inhibitor (DAS-181) targets the influenza virus receptor in the host respiratory tract and inhibits virus attachment. Cyanovirin-N (CVN) binds to high-mannose oligosaccharides on HA and inhibits the entry of virus into cells. For most influenza A viruses, the M2 ion channel blockers (amantadine and rimantadine) impede viral replication at an early stage of infection after penetration of the cell but prior to uncoating. M2 blockers inhibit the decrease in pH within the virion and thus block the release of viral RNA into the cytoplasm and prevent transportation of the viral genome to the nucleus. Inhibition of the viral polymerase, an essential component of viral RNA replication in the nucleus, can be blocked by the polymerase inhibitors ribavirin and T-705. Small interfering RNAs (siRNAs) that target different viral genes can inhibit viral replication. Several mechanisms of action have been proposed for the anti-influenza virus activity of ribavirin. One is the inhibition of the cellular enzyme inosine monophosphate dehydrogenase, resulting in a decrease in the intracellular guanosine 5’-triphosphate (GTP) that is required for nucleic acid synthesis. Ribavirin triphosphate also inhibits the function of virus-coded RNA polymerases which are necessary to initiate and elongate viral mRNAs. Late in infection, NA cleaves sialic acid–containing receptors and facilitates the release of budding virions. NA inhibitors (zanamivir, oseltamivir, peramivir, and LANI) block NA activity, preventing the release of virions from the cell.

There are currently few FDA-approved antiviral drug combinations, and more new antiviral drugs are urgently needed. Numerous attempts have been made to develop antiviral agents that target novel virus proteins or that act by novel mechanisms (Figure 2). Among these is the pyrazine molecule T-705 (favipiravir, 6-fluoro-3-hydroxy-2-pyrazinecarboxamide). The mode of action of ribosylated, triphosphorylated T-705 (T-705 RTP) is similar to that of ribavirin triphosphate: inhibition of influenza virus RNA polymerase [57,58]. Unlike ribavirin 5 monophosphate, T-705 RMP only weakly inhibits cellular inosine monophosphate dehydrogenase [58,59] and thus is less cytotoxic. These properties make T-705 a viable candidate influenza virus chemotherapy agent in humans. Phase II efficacy studies of T-705 were conducted in Japan during the 2007–2008 influenza season, and phase I studies were recently conducted in the United States. The benefits of using oseltamivir and T-705 in combination to treat H1N1, H3N2, and H5N1 influenza virus infection were recently demonstrated in a mouse model [60].

One factor in the unusual severity of disease caused by H5N1 influenza viruses is cytokine dysregulation, including rapid accumulation of proinflammatory cytokines (“cytokine storm”) after infection [61,62]. Therefore, inhibitors of inflammation are a possible therapeutic approach. It was shown that mice treated with a triple combination of the NA inhibitor zanamivir and inhibitors of inflammation, such as celecoxib and mesalazine, had a significantly better survival rate, survival time, and level of inflammatory markers than mice treated with zanamivir alone [63]. Many studies have shown that oxidative stress is important in the pathogenesis of pulmonary damage during influenza virus infections. Antioxidant molecules are therefore potentially useful against viral infection. It was shown that antioxidant molecule N-acetylcysteine (NAC) in combination with ribavirin enhanced survival of mice against a lethal influenza virus infection [64]. Further studies have demonstrated that treatment of mice with NAC combined with oseltamivir resulted in 100% survival [65], and thus suggest that antioxidant therapy increase survival by an improvement in host defense mechanisms, and/or by a direct antioxidant effect against oxidative stress associated with viral infection. In mice lethally challenged with A/Aichi/2/68 (H3N2) influenza virus the combination of an immunostimulatory bacterial preparation (cytoplasmic membranes of *Escherichia coli* WF stable protoplast type L-forms) with rimantadine resulted in synergistically increased protection, determined on the basis of virus lung titers, lung consolidation, mortality rates, protective indices, and survival times [66]. However, inclusion of novel principals for the treatment of human influenza virus infections, including H5N1 infection, require further development and accumulation of strong experimental evidence.

New formulations of conventional anti-influenza drugs and novel antiviral agents are currently at various stages of development. Parenteral administration of the NA inhibitors zanamivir (intravenous, IV) and peramivir (IV and intramuscular) are being evaluated in preclinical studies and clinical trials for the treatment of seasonal influenza A infection [67,68]. In Japan, peramivir has been licensed under the name Rapiacta® in 2010. Long-acting inhaled NA inhibitor (LANI) is a multimeric zanamivir compound (CS-8958) that persists longer in the lung and can be administered once weekly. These multimeric compounds have proven more potent than zanamivir and have provided the desired long-acting NA inhibition in a murine model of influenza infection, including infection with highly pathogenic H5N1 viruses [69,70]. Moreover, phase II clinical studies in Japan showed that a single inhaled dose of LANI was as effective as a standard five-day course of oseltamivir [71], and a double-blind, randomized, controlled trial showed that the LANI laninamivir octanoate was effective and well-tolerated in children with oseltamivir-resistant influenza A (H1N1) virus infection [72].

Advances in understanding the mechanisms of influenza virus replication have revealed a number of potential drug targets. The influenza virus receptor inactivator DAS181 (Fludase), a sialidase fusion protein, has shown inhibitory activity against a large number of seasonal influenza strains and a highly pathogenic avian influenza H5N1 strain [73,74]. Thus far, DAS181 has been well tolerated with no serious adverse events in phase I trials, and phase II trials have begun [75].

Other potential anti-influenza agents include Cyanovirin-N (CVN), a carbohydrate-binding protein that inhibits viral entry into cells by specifically binding to high-mannose oligosaccharides on the surface glycoproteins of enveloped viruses [76]; small interfering RNAs (siRNAs); immunomodulators; and immunotherapy with convalescent plasma and neutralizing antibodies.

## 6. Conclusions

The currently available influenza antivirals have a number of limitations; therefore, more effective antiviral strategies are needed. The possibilities include parenteral NA inhibitors, development of novel antiviral drugs (polymerase and endonuclease inhibitors, sialidases, long-acting NA inhibitors, hemagglutinin inhibitors, and non-structural protein NS1 inhibitors), and immunotherapy. Combination therapy with two or more drugs is a promising approach for control of seasonal influenza and of severe infection with highly pathogenic H5N1 influenza viruses. Drug combination studies *in vitro* and *in vivo* have shown the potential for synergistic or additive antiviral activity and for inhibiting the development of resistance; these qualities must now be demonstrated in clinical trials. All of the available information supports the initiation of clinical trials on combination chemotherapy for influenza. The planning of such studies is ongoing, and consideration is being given to clinical and virologic evaluations with determination of influenza virus loads in the patient, the molecular and biological characterization of viruses for resistance and fitness, and the detailed collection of pharmacokinetic data to evaluate safety and toxicity. Future considerations for combination therapy are dual NA inhibitors, triple combinations, and inclusion of novel agents. The new strategies directed at unexplored targets, such as drugs that affect host antiviral responses, should be also developed.

## Figures and Tables

**Table 1.** Effect of the double and triple drug combinations on influenza virus infections *in vitro*.

**Drug combinations**	**Influenza strain (subtype)**	**Method of analysis**	**Drug interactions**	**Ref.**
**Double combinations**				
**Rimantadine + Ribavirin**	A/Chick/Germany/27 (FPV Weybridge) (H7N7) ^a^	Virus yield inhibition (PFU)	Additive-synergistic	[22]
	A/USSR/77 (H1N1) A/Texas/77 (H3N2)	Virus yield inhibition (PFU)	Additive-synergistic	[26]
	A/England/80 (H1N1) ^b^ A/Aichi/68 (H3N2) B/Lee/40	Virus yield inhibition (PFU)	Subadditive-additive-synergistic	[27]
	A/Virginia/87 (H1N1) A/Virginia/88 (H3N2)	Virus yield inhibition (ELISA)	Additive	[34]
**Amantadine + Ribavirin**	A/USSR/90/77 (H1N1) A/Texas/1/77 (H3N2) A/New Jersey/76 (H1N1)	Virus yield inhibition (PFU)	Enhanced inhibitory effect	[26]
	A/Duck/MN/1525/81 (H5N1)	Virus yield inhibition (TCID_50_)	Synergistic ^c^	[38]
	*A/Duck/MN/1525/81 (H5N1)*^d^	Virus yield inhibition (TCID_50_)	No added benefit ^c^	[38]
	A/California/04/09 (H1N1) ^e^ A/California/05/09 (H1N1) ^e^ A/California/10/09 (H1N1) ^e^	Virus yield inhibition (NR staining)	Additive ^c^	[39]
**Zanamivir + Ribavirin**	A/Virginia/87 (H1N1) A/Virginia/88 (H3N2)	Virus replication inhibition (ELISA)	Additive	[34]
**Zanamivir + Rimantadine**	A/New Caledonia/20/99 (H1N1) A/Panama/2007/99 (H3N2)	Virus (TCID_50_, MDCK) and cell-associated yield inhibition (ELISA)	Additive-synergistic ^c^	[36]
**Oseltamivir carboxylate + Amantadine**	A/Nanchang/1/99 (H1N1) A/Panama/2007/99 (H3N2) A/Hong Kong/156/97 (H5N1)	Virus yield inhibition (PFU)	Enhanced inhibitory effect	[37]
	A/California/04/09 (H1N1) ^e^ A/California/05/09 (H1N1) ^e^ A/California/10/09 (H1N1) ^e^	Virus yield inhibition (NR staining)	Additive ^c^	[39]
	A/Duck/MN/1525/81 (H5N1)	Virus yield inhibition (TCID_50_)	Synergistic ^c^	[38]
	*A/Duck/MN/1525/81 (H5N1)*^d^	Virus yield inhibition (TCID_50_,)	No added benefit ^c^	[38]
**Oseltamivir carboxylate + Ribavirin**	A/California/04/09 (H1N1) ^e^ A/California/05/09 (H1N1) ^e^ A/California/10/09 (H1N1) ^e^	Virus yield inhibition (staining with NR)	Additive ^c^	[39]
**Oseltamivir carboxylate + Rimantadine**	A/New Caledonia/20/99 (H1N1) A/Panama/2007/99 (H3N2)	Virus (TCID_50_) and cell-associated yield inhibition (ELISA)	Additive-synergistic ^c^	[36]
**Peramivir + Rimantadine**	A/New Caledonia/20/99 (H1N1) A/Panama/2007/99 (H3N2)	Virus (TCID_50_) and cell-associated yield inhibition (ELISA)	Additive-synergistic ^c^	[36]
**Peramivir + Ribavirin**	A/NWS/33 (H1N1)	Virus yield inhibition (TCID_50_)	Synergistic ^c^	[35]
**Triple combinations**				
**Oseltamivir carboxylate + Amantadine + Ribavirin**	A/New Caledonia/20/99 (H1N1) A/Sydney/05/97 (H3N2) A/Duck/MN/1525/81 (H5N1)	Virus yield inhibition (TCID_50_), NR staining, RNA copies	Highly synergistic ^c^	[40]
	A/California/04/09 (H1N1) ^e^ A/California/05/09 (H1N1) ^e^ A/California/10/09 (H1N1) ^e^	Virus yield inhibition (NR staining)	Synergistic ^c^	[39]
	*A/New Caledonia/20/99(H1N1)^d^* *A/Wisconsin/67/05 (H3N2)^d^* *A/Duck/MN/1525/81 (H5N1)^d^*	Virus yield inhibition (NR staining)	Additive ^c^	[39]
	*A/Mississippi/3/01 (H1N1)^f^* *A/Hawaii/21/07 (H1N1)^f^*	Virus yield nhibition (NR staining)	Synergistic ^c^	[39]

**Note:** Drug interactions were evaluated based on inhibition of extracellular (in some experiments cell-associated) virus yield or virus replication in Madin Darby canine kidney (MDCK) cells (unless otherwise indicated). Unless indicated, the viruses were sensitive to all drugs used in the study. Abbreviations: Ref. = references; PFU = plaque forming units; TCID = tissue culture infectious dose; ELISA = enzyme linked immunosorbent assay; NR = neutral red.

a Studies were done in chick embryo fibroblasts cells.

b Studies were done in primary rhesus monkey kidney cells.

c Drug-drug interactions were analyzed by the three-dimensional model of Prichard and Shipman [33] using the MacSynergy^™^ II software program.

d Amantadine-resistant influenza virus variant (shown in italic).

e Alternative subtype designation – (H1N1 pdm 2009).

f Oseltamivir-resistant influenza virus variant (shown in italic).

**Table 2.** Antiviral activity of double drug combinations against influenza A and B viruses in a lethal mouse model.

**Drug combinations**	**Influenza strain (subtype), challenge dose/mouse**	**Drug treatment**	**Drug interactions**	**Ref.**
**Oseltamivir+ Amantadine**	A/Puerto Rico/8/34 (H1N1) ^a^	24 h ^b^	Enhanced protection	[45]
	A/Hong Kong/1/68 (H3N2) ^a^	24 h	Enhanced protection	[45]
	A/Vietnam/1203/04 (H5N1), 10 MLD_50_	24 h	Greater protection than monotherapy	[43]
	*A/Vietnam/1203/04 (H5N1)*^c^, 10 MLD_50_	24 h	No added benefit	[43]
	A/Duck/MN/1525/81 (H5N1), 10^4^ TCID_50_	4 h	Greater protection than monotherapy ^d^	[38]
	*A/Duck/MN/1525/81 (H5N1)*^c^, 10^5^ TCID_50_	4 h	No added benefit ^d^	[38]
**Oseltamivir + Rimantadine**	A/Quail/Hong Kong/G1/97 (H9N2) ^e^, 5 or 100 MLD_50_	4 h	Greater protection than monotherapy	[50]
	A/Aichi/2/68 (H3N2), 10 or 20 MLD_50_	4 h	Synergistic ^d^	[42]
**Oseltamivir + Ribavirin**	A/New Caledonia/20/99 (H1N1) ^e^, 10^6^ TCID_50_	24 h	No added benefit	[25]
	B/Sichuan/379/99 ^e^, 10^4^ TCID_50_	24 h	Synergistic	[25]
	A/Duck/MN/1525/81 (H5N1), 10^4^ TCID_50_	4 h	Greater protection than monotherapy ^d^	[38]
	*A/Duck/MN/1525/81 (H5N1)*^e^, 10^5^ TCID_50_	4 h	No added benefit ^d^	[38]
	A/Vietnam/1203/04 (H5N1), 5 MLD_50_	4 h, 8 days	Additive at some concentrations ^d^	[44]
	A/Turkey/15/06 (H5N1), 5 MLD_50_	4 h, 8 days	Additive at some concentrations ^d^	[44]
**Amantadine + Ribavirin**	A/Duck/MN/1525/81 (H5N1), 10^4^ TCID_50_	4 h	Greater protection than monotherapy ^d^	[38]
	*A/Duck/MN/1525/81 (H5N1)*^e^, 10^5^ TCID_50_	4 h	No added benefit ^d^	[38]
**Peramivir + Ribavirin**	A/NWS/33 (H1N1), 10^4^ TCID_50_	4 h	Additive-synergistic ^d^	[35]
**Oseltamivir + T-705**	A/NWS/33 (H1N1), 10^4^ TCID_50_	24 h	Strong synergy ^d^	[60]
	A/Victoria/3/75 (H3N2), 10^4^ TCID_50_	24 h	Strong synergy ^d^	[60]
	A/Duck/MN/1525/81 (H5N1), 10^4^ TCID_50_	2 h	Strong and minor synergy ^d^	[60]

**Note:** Drug interactions were evaluated on the basis of survival benefits provided by administration of drug combinations. Unless indicated, the viruses were sensitive to all drugs used in the study. Abbreviations: Ref. = references; MLD = mouse lethal dose; TCID = tissue culture infectious dose.

a Infectious virus was administered to mice using a 1–2 μm diameter particle aerosol in a Middlebrook Airborne Infection Apparatus.

b Unless indicated, the drugs were administered orally, twice daily for five days. Time indicates initiation of treatment before virus inoculation.

c Amantadine-resistant influenza virus variant (shown in italic).

d Drug-drug interactions were analyzed by the three-dimensional model of Prichard and Shipman [33] using the MacSynergy^™^ II software program.

e Mouse-adapted influenza virus variant.
